# Pneumonia

**Published:** 1905-10

**Authors:** E. S. Adams

**Affiliations:** Garrison, Texas


### Pneumonia.

GARRISON, TEXAS, 1905.

*Editor Texas Medical Journal:*

DEAR SIR: I hear so much said as to how little can be done for one suffering with pneumonia, that I am constrained to offer a few remarks on the subject. This applies to acute lobar pneumonia with which the average practitioner has so much to do. I think perhaps I am able to recognize a case of pneumonia and as I look at it there is one symptom that is pathogonomic of the disease.

We have rales that are crepetant and we have bloody sputa in other conditions than pneumonia. Well, say there is no doubt as to diagnosis and that we have a typical case of pneumonia, a febrile condition whose inception was a pronounced chill. In varying degrees we have delirium and other perturbations of function, under the expectant plan of treatment increases from day to day until a certain length of time has elapsed when the patient either succumbs or we have a crisis that marks a turning point and the patient improves. What is the cause of this perturbation of function? It is generally supposed to be due to the accumulation and retention of toxines in the system. What is it that kills in pneumonia? Aside from those hopeless cases where enough of lung substance is involved from the start to produce death from apnœa, the majority die from asthenia.

Before coming to the plan of treatment for which I claim no credit, as the medication is not at all original, I would like to say a few words about the pneumococcus, and I will say in passing that my evidence on this subject is entirely hearsay, as I have never seen one. I will say, however, that as a broad, general truth, most physicians with whom I have come in contact believe nature's processes to be conservative and beneficent, and if it is a fact that our buccal cavities always contain pneumococci, and that the accidental transmigration of these creatures to the proper spot would cause the development of pneumonia, I am bound to say that the idea grates very harshly on our conception of things. I would much rather feel that the pneumococcus was given to me by a beneficent Creator for a good purpose, and if I should ever be so unfortunate as to develop pneumonia, He would be on the ground to act as a scavenger and aid in removing the trouble. Well, now for the treatment:

Let the dominant idea be to force a condition of things identical with a favorable crisis. Under the expectant plan of treatment, along towards the ninth day of the disease, on your visit to your patient you can not say whether you will find him "in articulo mortis," or whether you will find him bathed in a cool perspiration, fever all gone, pulse regular, though "weak and wabbly," with a "look out of his eyes" that says as plainly as spoken, "I've beaten death to the goal." Can we force a condition of this kind sooner than the "expectant idea" would look for it? I contend that we can and we can do it with sodium salicylate superior. Sodium salicylate made from the oil of wintergreen does not possess the depressing properties of that made synthetically. Why this is, I can not say, unless it is due to the fact that nature can beat man doing things. Take the average case of one-lunged pneumonia and give antiseptic (alterative if you please) doses of calomel till you get healthy bile (nature's antiseptic) in the discharges, and give sodium salicylate superior to force a condition of things similar to what occurs in a favorable crisis, sustaining the patient with one-sixtieth to one-thirtieth grain of strychnine every four hours, I believe you will experience the feeling of having cured your patient as much so as you would had you given enough quinine to meet a malarial paroxysm. If ten grains every four hours fail, try fifteen and up to twenty. In other words, force the crisis and save your patient, and do not sit idly by and allow toxines in your patient's system to kill him when you can certainly neutralize or eliminate the death-dealing factor with the remedy named.

Respectfully,

E. S. Adams.

